# Experimental Type 2 Diabetes Differently Impacts on the Select Functions of Bone Marrow-Derived Multipotent Stromal Cells

**DOI:** 10.3390/cells10020268

**Published:** 2021-01-29

**Authors:** Jonathan Ribot, Cyprien Denoeud, Guilhem Frescaline, Rebecca Landon, Hervé Petite, Graciela Pavon-Djavid, Morad Bensidhoum, Fani Anagnostou

**Affiliations:** 1 Université de Paris, CNRS, INSERM, B3OA, 75010 Paris, France; jonathan.ribot@outlook.fr (J.R.); cyprien.denoeud@gmail.com (C.D.); frescaline@anrt.asso.fr (G.F.); rebecca.landon@inserm.fr (R.L.); herve.petite@univ-paris-diderot.fr (H.P.); morad.bensidhoum@paris7.jussieu.fr (M.B.); 2 INSERM U1148, Laboratory for Vascular Translational Science, Cardiovascular Bioengineering, Université Sorbonne Paris Nord, 93430 Villetaneuse, France; graciela.pavon@univ-paris13.fr; 3 Department of Periodontology, Service of Odontology–Pitié Salpêtrière Hospital, AP-HP et U.F.R. of Odontology, 75013 Paris, France

**Keywords:** diabetes type 2, MSCs, stem cell, ZDF, bone marrow

## Abstract

Bone marrow-derived multipotent stromal cells (BMMSCs) represent an attractive therapeutic modality for cell therapy in type 2 diabetes mellitus (T2DM)-associated complications. T2DM changes the bone marrow environment; however, its effects on BMMSC properties remain unclear. The present study aimed at investigating select functions and differentiation of BMMSCs harvested from the T2DM microenvironment as potential candidates for regenerative medicine. BMMSCs were obtained from Zucker diabetic fatty (ZDF; an obese-T2DM model) rats and their lean littermates (ZL; controls), and cultured under normoglycemic conditions. The BMMSCs derived from ZDF animals were fewer in number, with limited clonogenicity (by 2-fold), adhesion (by 2.9-fold), proliferation (by 50%), migration capability (by 25%), and increased apoptosis rate (by 2.5-fold) compared to their ZL counterparts. Compared to the cultured ZL-BMMSCs, the ZDF-BMMSCs exhibited (i) enhanced adipogenic differentiation (increased number of lipid droplets by 2-fold; upregulation of the Pparg, AdipoQ, and Fabp genes), possibly due to having been primed to undergo such differentiation in vivo prior to cell isolation, and (ii) different angiogenesis-related gene expression in vitro and decreased proangiogenic potential after transplantation in nude mice. These results provided evidence that the T2DM environment impairs BMMSC expansion and select functions pertinent to their efficacy when used in autologous cell therapies.

## 1. Introduction

Type 2 diabetes mellitus (T2DM) has reached epidemic proportions worldwide. In addition to macro- and micro-vascular complications such as cardiovascular diseases, retinopathy, neuropathy, nephropathy, and prolonged/incomplete wound healing, T2DM is associated with increased bone fragility [1] and impaired bone healing [2,3,4]. Specifically, in the bone marrow, T2DM induces microvascular rarefaction and vascular endothelial cell dysfunction [5], reduces osteoblastogenesis, and increases adiposity [6,7]. These conditions have also been implicated in changing the stem cell niche, resulting in a compromised function of the bone marrow-derived multipotent stromal cells (BMMSCs)/mesenchymal stem cells (MSCs).

BMMSCs are a unique type of adult stem cells. They home in on the sites of injuries and contribute to tissue repair [8], as well as playing a key role in regulating new blood vessel formation and function [9]. BMMSCs exhibit high proliferation as they differentiate into various cells types, including fibroblasts, osteoblasts, chondrocytes, adipocytes, and vascular endothelial cells [10]. They also exert paracrine effects by releasing growth factors and cytokines [11]. The phenotype of these cells is strongly affected by their “milieu”. Diabetic microenvironment encompassing high glucose, inflammation, hypoxic conditions, and Reactive Oxygen Species (ROS) content has deleterious effects on the functionality of BMMSCs [12]. More precisely, in the case of obese T2DM, however, the data are inconclusive and incomplete to date regarding the BMMSC phenotype and functional properties [13,14]. Moreover, owing to their multipotency and ability to secrete angiogenic factors, cytokines, and immunomodulatory chemical compounds, BMMSCs are an attractive cell source to treat the secondary complications of T2DM [15,16]. Nevertheless, in preclinical and clinical studies, autologous use has resulted in mixed outcomes [17]. Despite the fact that the T2DM milieu is a critical aspect in preparing BMMSCs from diabetic donors and in predicting their function after implantation, relatively little is known about the consequences of T2DM for the BMMSC functions which are needed for the success of pertinent clinical studies.

The present study focused on elucidating select functions and the differentiation potential of BMMSCs harvested from the T2DM microenvironment in order to improve understanding of induced alterations and to establish the potential of these cells as candidates for regenerative medicine applications. To this end, BMMSCs were obtained from Zucker diabetic fatty (ZDF) (Leprfa/fa) rats (an obese T2DM well defined animal model) [18,19], which exhibit musculoskeletal [20] and vascular [4,21,22] complications, and from their Zucker lean (ZL) littermates (respective healthy controls).

## 2. Materials and Methods

### 2.1. Animal Model

Adult 8-week-old male, Swiss, nude mice and 13-week-old male, obese Zucker fa/fa rats (ZDF) and their lean fa/+ littermates (ZL) were purchased from Charles River (L’Arbresle, France). The animals were housed, operated, and euthanized based on the procedures in accordance with the “European Community Standards on the Care and Use of Laboratory Animals”. The experimental procedures used in the present study were approved by the Ethics Committee of the Paris-Diderot University (N°01610.01/S69), Paris, France.

The diabetic conditions in the aforementioned rats were determined on the day of sacrifice. Venous blood was collected and the glycemic state was evaluated using a glucometer (Roche Diagnostics, Meylan, France). The glucose and fructosamine concentrations were determined in the collected blood plasma using commercially available kits from Roche Diagnostics (Roche Diagnostics, France) and following the manufacturer’s instructions.

### 2.2. Isolation of Rat Bone Marrow-Derived Multipotent Stromal Cells

BMMSCs were obtained as previously described [23]. Briefly, the femurs and tibiae from each rat were cleaned of connective tissues and their respective epiphyses were removed to allow the insertion of a 23-gauge needle connected to a syringe containing alpha-Modified Eagle’s Medium (αMEM) (Invitrogen, Cergy Pontoise, France). Harvested cells were then homogenized in complete culture medium (αMEM) supplemented with 10% (*v*/*v*) fetal calf serum (FCS) and 1% (*v*/*v*) antibiotic/antimycotic (ATB/ATM) solution (PAA Laboratories GmbH, Pasching, Austria). The isolated cells from ZL and ZDF rats were seeded separately at a density of 5 × 10^5^ cells/cm^2^, and cultured at 37 °C in a humidified 5% CO_2_/95% air environment, and the supernatant media (containing non-adherent cells) were discarded after 2 days of culture. Fibroblastic colonies (CFU-Fs) were formed at day 5 of culture and all pooled at day 12 (cell passage 1). For amplification of the ZL- and ZDF-BMMSC populations, the cells at a subconfluent stage (after 3 days of culture) were trypsinized using a 0.005% trypsin/EDTA (Ethylenediaminetetraacetic acid) solution and seeded at 10 × 10^3^ cells/cm^2^. During cell culture, the supernatant media were replaced twice a week. BMMSCs (passages 2–3) were used for all the experiments of the present study. The BMMSC origin was assessed using fluorescence-activated cell sorting and monitoring for expression of the CD29 (clone Hmb1–1; eBioscience), the CD45 (clone OX1; eBioscience), and the CD90 markers (clone HIS51; Life Technology), as well as their differentiation into the osteogenic and adipogenic lineages.

### 2.3. Fibroblastic Colony-Forming Unit (CFU-F) Assay

After being harvested from bone marrows, mononuclear cells were seeded at 5 × 10^5^ cells/cm^2^ in individual wells of 6-well-tissue-culture plates. After 4 h, the supernatant culture medium (containing non-adherent cells) was discarded and the adherent cells were cultured for 14 days. These cells were then rinsed with phosphate buffered saline (PBS) and stained in situ using the May-Grünwald Giemsa protocol. The stained cells in each well were photographed using a Nikon Eclipse TE 2000U inverted microscope. Cell colonies measuring more than 2 mm in diameter (which also contained more than fifty adherent fibroblastoid cells), but not smaller cell clusters, were further analyzed. The shape of each colony was visualized, and the respective surface area was automatically marked using the NIS-Elements BR 2.30 computer image analysis system (Nikon Instruments Inc., Melville, NY, USA). For each condition tested, the colony forming efficiency (CFE) was calculated as the total number of CFU-Fs per well-formed 10^6^ cells seeded.

### 2.4. Cell Adhesion

ZDF- and ZL-BMMSCs in complete culture medium were separately seeded at 8.5 × 10³ cells/cm^2^ in individual wells of 12-well cell culture plates (TPP, Trasadingen, Switzerland). After 15, 30, 45, 60, 90, 120, 180, and 240 min, the supernatant media were removed. The adherent cells were fixed using a methanol–PBS (1/1 *v*/*v*) solution (Prolabo) and then stained with Giemsa solution (Sigma-Aldrich St. Louis, MO, USA). Six random spots per well surface area were photographed using light microscopy (Keyence VHX100, Osaka, Japan). Afterward, the adherent cells were counted using the data processing software Image J (National Institute of Health, Bethesda, MD, USA).

### 2.5. Cell Proliferation

ZDF- and ZL-BMMSCs in complete culture medium were each seeded at the density of 3 × 10^3^ cells/cm^2^ in individual wells of 12-well cell culture plates. Following cell adhesion, BMMSC proliferation was determined daily for up to seven consecutive days of culture. The cells present in each well were fixed using 95% ethanol at the prescribed time points, stained with propidium iodide (PI; 50 μg/mL; Sigma-Aldrich) at 37 °C for 30 min, and counted using flow cytometry analysis (Attune NxT Flow Cytometer, Thermo Fisher Scientific, Waltham, MA, USA).

### 2.6. Apoptosis

BMMSCs derived from ZL and ZDF rats (passage 2) were cultured in serum-free αMEM for 48 h. The percentage of apoptotic cells was then determined using Guava^®^ Nexin Reagent and flow cytometry analysis according to the manufacturer’s instruction.

### 2.7. Cell Migration

BMMSC migration was determined using commercially available 24-well Boyden chambers (Corning Costar, Cambridge, MA, USA) whose two compartments were separated with porous polycarbonate membranes with 8 µm diameter pores. Briefly, either ZDF or ZL cells (75 × 10^3^ cells/100 µL) in serum-free αMEM (migration medium) were placed in the upper chamber of each Boyden chamber. The bottom chamber of each Boyden system contained 0.6 mL of complete culture medium. The cell migration experiments were conducted in a humidified, 37 °C, 5% CO_2_/95% air environment, overnight. The cells that had traversed, but still adhered to the other side of the membrane separating the two Boyden compartments, were fixed using 4% paraformaldehyde and then stained in situ with the May Grunwald-Giemsa stain. Each membrane was then excised, mounted on a glass slide, and visualized using light microscope. The cells present on four randomly-chosen, separate areas per membrane were counted in situ. Pertinent numerical data were averaged and reported “as cell numbers per test”.

### 2.8. Reactive Oxygen Species (ROS) Release

To measure ROS production in ZDF- and ZL-BMMSCs, the DCFH-DA (2′,7′-Dichlorofluorescin diacetate) (DCFH-DA, Thermo Fischer Scientific, Waltham, MA, USA), an oxidation-sensitive indicator, was used. ZDF- and ZL-BMMSCs in complete culture medium were separately seeded at 1 × 10³ cells/cm^2^ in individual wells of 96-well cell culture plates. After 12 h, the cells were rinsed and incubated with 100 µL of 5 µm 2′7′ diacetate dichlorofluoresceine at 37 °C for 1 h. Oxidative stress was induced by adding 50 µL of tert-butyl peroxide 400 µM (Luperox^®^, Sigma–Aldrich, St. Louis, MO, USA) to the supernatant medium of each well. Fluorescence (excitation wavelength of 485nm and emission wavelength of 530nm) of each supernatant sample was monitored every 5 min for 40 min using a spectrophotometer (TECAN Infinite^®^ 200 PRO Series instrument, Männedorf, Switzerland). Fluorescence was directly correlated to ROS release by the ZDF- and ZL-BMMSCs.

### 2.9. Differentiation Assays

#### 2.9.1. Adipogenic Differentiation

ZDF- and ZL-BMMSCs in complete medium were separately seeded at 10 × 10^3^ cells/cm^2^ in individual wells of 12-well cell culture plates and cultured under standard cell culture conditions until they reached 90% confluency. The supernatant media of these cell cultures were replaced with either complete medium (used as control) or adipogenic medium (i.e., complete medium supplemented with 100 ng/mL biotin, 250 nM dexamethasone, 0.5 mM isobutyl methylxanthine, 60 µM indomethacin, and 10 µg/mL insulin; all chemicals were from Sigma-Aldrich). All supernatant media were replaced every 2 days of cell culture. Adipogenic differentiation was determined by monitoring the expression of select adipogenic markers (specifically, Pparg, AdipoQ, and Fabp4) using quantitative polymerase chain reaction (qRT-PCR), and by the formation of intracellular lipid droplets detected using Oil Red-O staining.

#### 2.9.2. Osteogenic Differentiation

BMMSCs in complete medium were seeded at 10 × 10^3^ cells/cm^2^ in individual wells of 12-well cell culture plates and cultured under standard cell culture conditions until they reached 80% confluency. The supernatant media were then replaced with either complete culture medium (used as control) or osteogenic-induction medium (Lonza France, Levallois). The supernatant media were replaced every 2 days of cell culture. Osteogenic differentiation was determined by monitoring the expression of select osteogenic markers (specifically, Runx2, CollI, and Osterix) using qRT-PCR, by the presence of calcium-containing mineral deposits (stained with Alizarin Red) in the extracellular matrix of the cultured BMMSCs, and by determination of ALP (Alkaline phosphatase) activity.

#### 2.9.3. Angiogenic Differentiation and Potential

ZDF- and ZL-BMMSCs were seeded at 10 × 10^3^ cells/cm^2^ in individual wells of 12-well cell culture plates and cultured under standard cell culture conditions for 24 h. The supernatant media were then replaced with either culture medium supplemented with 2% FCS (*v*/*v*) (used as control) or angiogenic-induction medium composed of αMEM, 2% FBS (Fetal bovine serum) (*v*/*v*), and 50 ng/mL VEGF (Vascular endothelial growth factor) (all chemicals were from Sigma-Aldrich). The BMMSCs were cultured under those conditions for 14 consecutive days. The supernatant media were replaced every 2 days of cell culture. Endothelial differentiation was determined by monitoring the expression of select angiogenic markers (specifically *Vegf*, *Fgf2*, *Hif1*, and *Hif2*) using qRT-PCR. In addition, the formation of tubular structures was determined upon culturing the aforementioned cells on Matrigel for 24 h.

### 2.10. Matrigel Plug Assay

BMMSCs from either ZL or ZDF rats (10^6^ cells in 500 µL of Matrigel) were implanted subcutaneously into the flank of 8-week-old, Swiss, nude mice. After 14 days, the mice were euthanized using Dolethal (Vétoquinol). Each Matrigel plug was excised, placed in 500 µL RIPA buffer (Radioimmunoprecipitation assay buffer), and processed using a Retsch MM 300 TissueLyser at 30 pulses/min for 2 min. The hemoglobin content in each excised Matrigel implant was determined using a commercially available assay (Hemoglobin Colorimetric Assay kit; Cayman).

### 2.11. Quantitative Polymerase Chain Reaction (qRT-PCR)

Expression of select osteogenic and adipogenic markers by BMMSCs cultured in either adipogenic, osteogenic, or angiogenic media was determined using total RNA extracted from BMMSCs pre-treated with Trizol reagent (Life Technologies) according to the manufacturer’s protocol. The RNA integrity and purity/concentration were checked using spectrophotometry (Nanodrop 1000, Labtech, Palaiseau, France). Random-primed cDNA was synthesized from 1 µg of total RNA using the SuperScript™ II RT Kit (Invitrogen). Equal volumes of cDNA were used to program amplifications through real-time PCR (RT-PCR). qRT-PCR was performed using an iCycler thermocycling apparatus (MyiQ™ Single-Color RT-PCR; Bio-Rad Laboratories, Marnes-la-Coquette, France) in the presence of TaqMan Universal PCR Master Mix (Applied Biosystems) and the following specific primers: for osteogenic differentiation: Runt-related transcription factor 2 (Runx2), Collagen type I (collI), and Osterix; for adipogenic differentiation: Peroxisome proliferator-activated receptor gamma (Pparg), Adiponectin (AdipoQ), and fatty acid binding protein 4 (Fabp4); and for endothelial differentiation: analysis of the involved angiogenic factors was performed using a multiplex containing 84 angiogenic-related genes (and the rat angiogenesis RT^2^ Profiler PCR Array; Qiagen, Hilden, Deutschland). Briefly after the activation of DNA polymerase at 95 °C for 10 min, cDNA was amplified by performing 40 two-step PCR cycles: a 15-s denaturation step at 95 °C, followed by a 60-s annealing, and an extension step at 60 °C. Each sample tested was run in triplicate. All data were subsequently analyzed using MyiQ™ Software (Bio-Rad Laboratories, Marnes-la-Coquette, France). Quantitation of gene expression was performed using the comparative threshold cycle method (∆∆Ct).

### 2.12. Statistical Analyses

Each experiment was performed in triplicate and at least three separate times using different cell preparations. Numerical data were reported as mean ± standard error of the mean (SEM). For time-dependent experiments, analysis of variance (ANOVA) was used for unpaired two-tailed samples, and the experimental data were compared to their respective controls. For time-independent experiments, Student’s t-tests were used for unpaired two-tailed samples, and the experimental data were compared to their respective controls. *p* values less than 0.05 were considered statistically significant.

## 3. Results

### 3.1. Animals

At 13 weeks, the ZDF rats had significantly (*p* < 0.001) higher body weight compared to their age-matched ZL rats (specifically, 339.2 ± 4.61 vs. 404.2 ± 13.07, respectively; data not shown). Compared to the ZL rats, the ZDF rats also had significantly (*p* < 0.05) increased serum glucose and fructosamine levels (specifically, 2.26-fold and 1.52-fold, respectively; data not shown).

### 3.2. T2DM Affects the Number of Bone Marrow Mononuclear Cells and Select Functions of the Expanded BMMSCs

**Figure 1:**
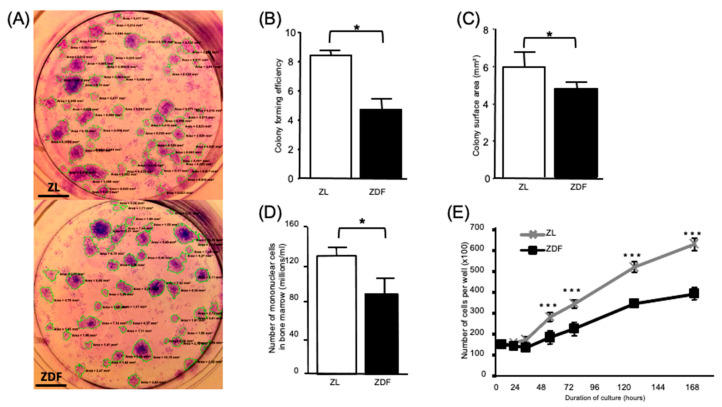
Type 2 diabetes mellitus (T2DM) affects the number, clonogenicity, and proliferation of cultured bone marrow-derived multipotent stromal cells (BMMSCs). Formation of fibroblastic colonies (CFU-Fs) in complete medium was assayed using bone marrow mononuclear cells from diabetic (ZDF) and non-diabetic (ZL) rats. (**A**) Representative images of CFU-F colonies, Scale bar = 0.5 cm (**B**) The colony forming efficiency (CFE), (**C**) The average area of each colony shown in Figure 1A, (**D**) The number of mononuclear cells present in the collected bone marrow, counted after isolation of the BMMSCs from two tibiae and two femurs per rat (n = 3), and (**E**) The number of ZDF-BMMSCs in alpha-Modified Eagle’s Medium (αMEM) containing 10% Fetal bovine serum (FBS), exhibiting lower proliferation over a 7-day period of culture. Values are mean ± standard error of the mean (SEM). The data are from 3 independent wells per condition tested in 3 independent experiments (n = 9). * *p* < 0.05; *** *p* < 0.001.

The formation of CFU-Fs was significantly (*p* < 0.05) lower (specifically, by 2-fold) in the BMMSCs harvested from the ZDF than from the ZL rats (Figure 1A,B). The average colony size formed by BMMSCs from the diabetic ZDF animals was significantly lower (by 20%) compared to that obtained with the cells from non-diabetic ZL animals (Figure 1C). The number of mononuclear cells harvested from the bone marrow of diabetic (ZDF) animals was significantly (*p* < 0.05) lower (by 30%) than the cells from non-diabetic (ZL) rats (Figure 1D). The MSC markers CD90 and CD105 were similarly expressed by passage-2-expanded cells from both ZDF and ZL rats. None of the cell types expressed the leukocyte marker CD45 (data not shown). Expression of the aforementioned MSC markers was similar for the cells harvested from the ZDF and ZL rats. After culture under standard conditions for up to 7 days, the proliferation of BMMSCs from the ZDF rats was significantly (*p* < 0.001) less than that observed for the BMMSCs from the ZL animals (Figure 1E).

**Figure 2:**
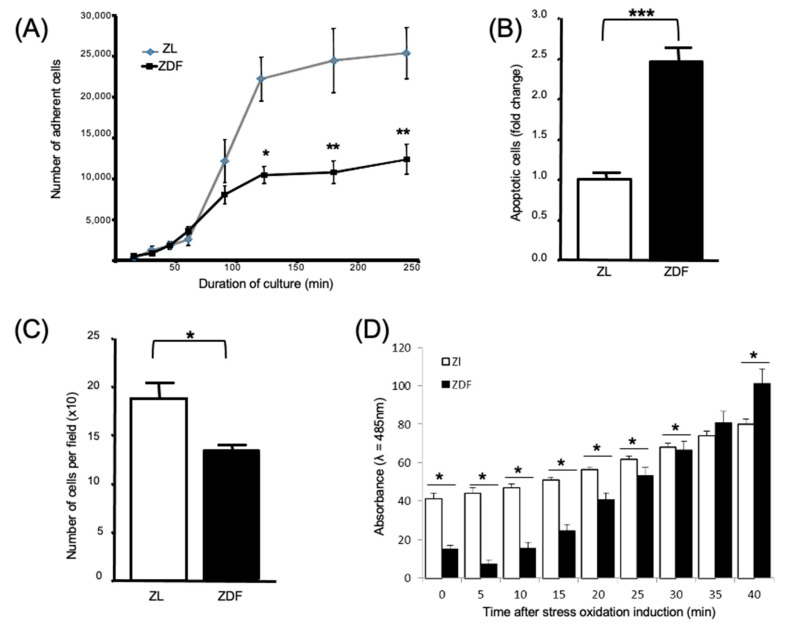
Impaired apoptosis, migration, adhesion, and Reactive Oxygen Species (ROS) production of ZDF-BMMSCs. (**A**) BMMSCs from ZDF and ZL rats were each seeded in 12-well culture plates, fixed using methanol–phosphate buffered saline (PBS) solution, and stained with Giemsa solution. Adherent cells were counted using the data processing software Image J. (**B**) Flow cytometry analysis of apoptosis using cells cultured under serum-free conditions for 48 h and stained with annexine-PI, (**C**) Cell migration was determined using commercially available 24-well Boyden chambers. Cells present on four randomly-chosen, separate areas per membrane were counted in situ. (**D**) Oxidative stress was induced by adding tert-butyl peroxide, then light absorbance (λ = 485 nm) was measured every 5 min for 40 min. Values are mean ± SEM. The data are from 3 independent wells per condition tested in 3 independent experiments (n = 9). * *p* < 0.05; ** *p* < 0.01; *** *p* < 0.001.

The number of expanded ZDF-BMMSCs adhered to tissue culture polystyrene 2 and 4 h after seeding was significantly (*p* < 0.01) lower (by 45%) than the respective results obtained with the ZL-BMMSCs (Figure 2A). BMMSCs from ZDF rats (which had been cultured in serum-free media for 2 days and then double-labeled with annexin/PI (propidium iodide)) exhibited a significantly (*p* < 0.001) higher level of apoptosis (specifically by 2-fold) than the BMMSCs from the ZL animals (Figure 2B). These results provided evidence that, compared to the BMMSCs from the diabetic ZDF rats, the cells from the non-diabetic ZL rats are more sensitive to serum-deprivation. In terms of their chemotactic capability, the BMMSCs from the ZDF rats exhibited significantly (*p* < 0.001) lower migration (by 25%) than the cells from the ZL control animals (Figure 2C). Furthermore, compared to the results obtained from ZL-BMMSCs, a different profile was observed for the ZDF-BMMSCs in terms of the kinetics of ROS production (Figure 2D).

### 3.3. T2DM Differently Affects the Differentiation Potential of the In Vitro BMMSCs

**Figure 3:**
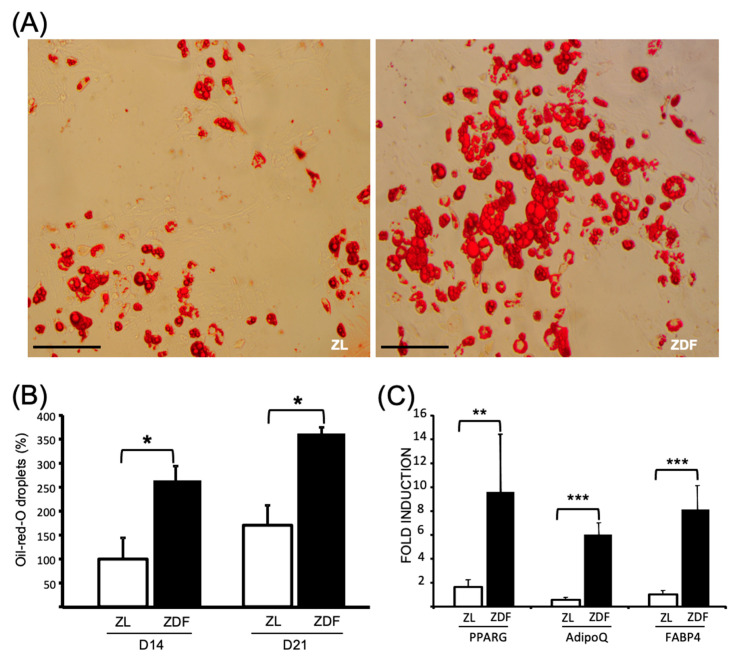
Enhanced adipogenic differentiation of the ZDF-BMMSCs. (**A**) Representative light microphotograph of BMMSCs from ZDF and ZL rats cultured in adipogenic-induction medium for 21 days, Scale bar = 200 μm. (**B**) Oil red-O droplets in BMMSCs after 14 and 21 days of culture measured by spectrophotometer, (**C**) Expression levels of the *Pparg*, *AdipoQ*, and *Fabp4* genes in the BMMSCs measured by qRT-PCR at day 14. Values are mean ± SEM. The data are from 3 independent wells per condition in 3 independent experiments (n = 9). * *p* < 0.05; ** *p* < 0.01; *** *p* < 0.001.

The potential of ZDF- and ZL-BMMSCs to undergo adipogenic differentiation was assessed by culturing these cells under normoglycemic conditions in adipogenic medium for 14 and 21 consecutive days. Under these conditions, the BMMSCs from the ZDF rats contained a significantly (*p* < 0.05) higher number of triglyceride droplets than the BMMSCs from the ZL animals (Figure 3A,B). In addition, analysis of the chosen, select adipogenic markers revealed upregulation of Pparg (by 10-fold), AdipoQ (by 6-fold), and Fabp4 (by 8-fold) in the ZDF-BMMSCs (Figure 3C).

**Figure 4:**
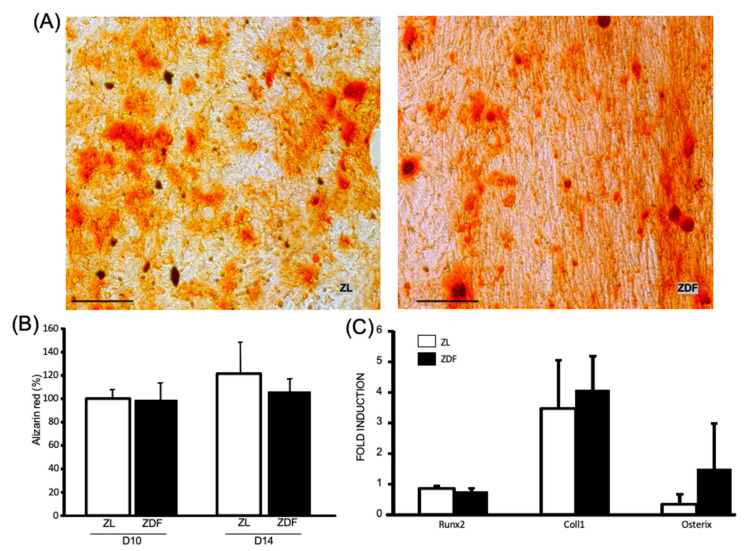
Osteogenic differentiation of ZDF- and ZL-BMMSCs. BMMSCs from ZDF and ZL rats were submitted to an osteogenic induction medium. (**A**) Photograph of Alizarin red staining after 21 days of culture in osteogenic medium, (**B**) Dosage of alizarin red staining by spectrophotometry, Scale bar = 200 μm. (**C**) Expression levels of the *Runx2*, *CollI*, and *Osterix* genes in the BMMSCs measured by qPCR at day 14. Values are mean ± SEM, calculated from 3 independent wells per condition in 3 independent experiments (n = 9)

The potential of ZDF- and ZL-BMMSCs to undergo osteogenic differentiation was assessed by culturing these cells in osteogenic medium for 14 and 21 consecutive days. As evidenced by the results obtained following calcium and alizarin red staining (Figure 4A,B, respectively), after 21 days of cell culture the osteogenic differentiation exhibited by ZDF- and ZL- BMMSCs was similar. The transcription factors *Runx2*, *CollI*, and *Osterix* were also similarly expressed by the BMMSCs from the ZL and ZDF rats after 14 days of culture (Figure 4C).

### 3.4. T2DM Reduces the BMMSC Angiogenic Potential

**Figure 5:**
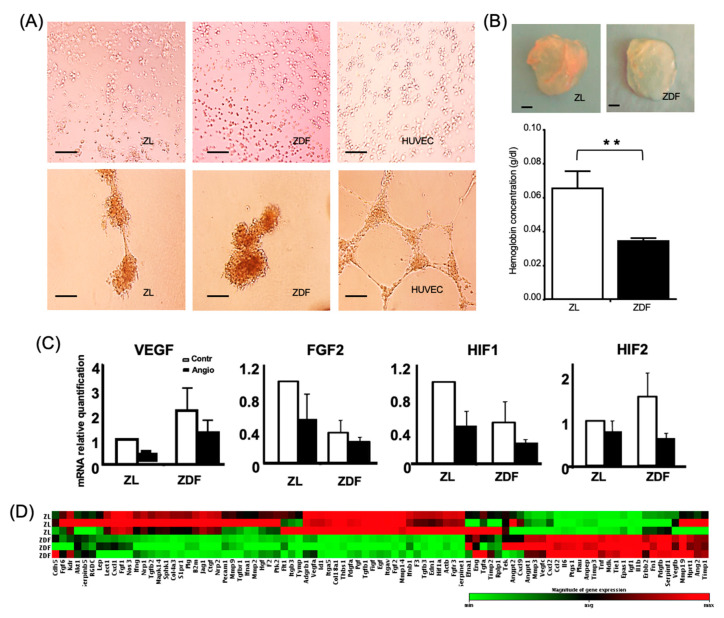
ZDF-BMMSCs reduced ZL- are more angiogenesis inducing than their counterpart. (**A**) Light micrographs of ZL-, ZDF-BMMSCs, and HUVECs (Human Umbilical Vein Endothelial Cells) cultured in an angiogenic medium for a 14-day period and then on a Matrigel for 24 h, Scale bar = 100 µm (**B**) Light micrograph of explanted Matrigel plugs seeded with ZL- and ZDF-BMMSCs (upper panel) and hemoglobin content of those plugs (lower panel) after 14 days implantation in nude mice, Scale bar = 1 mm (**C**) Expression of *VEGF, FGF2, HIF1*, and *HIF2* from ZL and ZDF-BMMSCs cultured with control (white) or angiogenic induction medium (black), (**D**) Expression of select angiogenic genes in ZDF-BMMSCs relative to the results obtained from ZL-BMMSCs under the same normoglycemic conditions of culture after 14 days, via quantitative PCR. Values are mean ± SEM. n = 3. ** *p* < 0.01. ZL-BMMSCs (i.e., from control rats). ZDF-BMMSCs (i.e., from diabetic rats).

BMMSCs from the ZL and ZDF rats did not differentiate into endothelial cells (Figure 5A). Expression of the chosen, select angiogenic markers, *VEGF*, *FGF2*, *HIF1*, and *HIF2* from ZL and ZDF cells exposed or not to angiogenic medium did not exhibit significant differences (Figure 5C). Moreover, and in contrast to the results obtained with HUVECs (Human Umbilical Vein Endothelial Cells, positive control), no tubular structures were formed when the aforementioned cells were cultured on Matrigel for 24 h (Figure 5A). To determine the proangiogenic potential of the BMMSCs from ZDF and ZL rats in vivo, the model of implanted Matrigel plugs was used. Upon implantation of the ZL- and ZDF-BMMSCs seeded in Matrigel plugs in the flank of nude mice for 14 days, the ZDF-BMMSC-containing gels exhibited less angiogenesis than the respective gels containing ZL-BMMSCs. Supporting evidence is provided by the illustration of representative explanted plugs in Figure 5B: the color of the plug seeded with ZDF-BMMSCs is pale, but the one seeded with ZL-BMMSCs is red (evidence of higher blood vessel content). Compared to the ZL-BMMSC-containing specimens, the Matrigel plugs originally seeded with ZDF-BMMSCs exhibited a significant (*p* < 0.001) decrease (by 2-fold) in their hemoglobin content (Figure 5B). Additional evidence corroborating the observed differences was provided by the results regarding the expression of known angiogenesis-related genes in the ZL- and ZDF-BMMSCs cultured under normoglycemic conditions before their seeding on Matrigel plugs. The results of multiplex analysis of 84 genes tested showed that ZDF-BMMSCs differentially expressed antiangiogenic and angiogenic genes (Figure 5D). Specifically, and compared to pertinent results obtained with ZL-BMMSCs, eleven anti-angiogenic genes, including *Col18a1* (by 3-fold), *Col4a3* (by 8-fold), *F2* (by 2-fold), *Infg* (by 2-fold), *Itga5* (by 3-fold), *Jag1* (by 2-fold), *Pdgfa* (by 2-fold), *S1pr1* (by 3-fold), *Serpine1* (by-3 fold), *TGFb1* (by 3-fold), and *TGFb3* (by 2-fold) were significantly (*p* < 0.05) down-regulated in the ZDF-BMMSCs, while ten pro-angiogenic genes, including *Anpep* (by 3-fold), *MCP-1* (by 3-fold), *Mip-2* (by 3-fold), *IGF-1* (by 5.5-fold), *IL-6* (by 2-fold), *PLAU* (by 2-fold), *Tie1* (by 5.5-fold), and *TNF* (by 4-fold) were significantly (*p* < 0.05) up-regulated in the ZDF-BMMSCs. It should be noted that the observed difference in gene expression level did not affect the lack of differentiation of the BMMSCs into the endothelial lineage in vitro.

## 4. Discussion

Considering that bone marrow mesenchymal stem cells have the capacity to proliferate, differentiate into several lineages, and home in on the sites of injury, they represent a very promising but still untapped cell source for the treatment of impaired diabetic wounds [16]. Published research findings regarding the effects of T2DM conditions on BMMSCs’ functional capacity are few and not conclusive [12,14]; moreover, selection between autologous and allogeneic cells is yet to be discussed [13,17]. These gaps in the current knowledge about T2DM provided motivation and justification for the present comparative study of the differentiation and select functions of BMMSCs from ZDF 13/14-old rats, a model for the short-term, diabetic condition [20], and their ZL littermates as controls. The results provided evidence that BMMSCs harvested from the T2DM bone marrow microenvironment differed from BMMSCs harvested from the non-diabetic milieu as follows: (i) there were fewer cells; (ii) they exhibited impaired functionality but increased adipogenic differentiation when cultured under normoglycemic conditions; and (iii) they had decreased proangiogenic potential after transplantation in a non-diabetic milieu. These results highlight the intrinsic dysfunction of T2DM-BMMSCs and have implications for the potential use of these cells in autologous cell therapies and in tissue engineering.

The limitation in the number of mononuclear cells harvested from the ZDF bone marrow is in line with other literature reports regarding T2DM in (db/db) mice [24]. This may be explained by the depletion of resident stem/progenitor cells due to the disturbed stem cell niche in the bone marrow of the diabetic animals [5,6]. A consequence of the reduced number of BMMSCs may be the diabetes-associated impaired repair of injured tissues [8,24]. Most importantly, compared to ZL-BMMSCs, the ZDF-derived cells exhibited altered functions even when they were cultured ex vivo under normoglycemic conditions. Specifically, ZDF-BMMSCs’ migration, clonogenicity, and proliferation ability were significantly (*p* < 0.05) decreased, while their apoptosis was increased compared to the respective results obtained with ZL-BMMSCs. These results imply that expansion of T2DM-BMMSCs to reach to sufficient numbers for autologous transplantation is challenging and that these cells may have reduced potential to promote tissue healing when they are administered in autologous cell-therapies.

The reasons for which cultured ZDF-BMMSCs exhibited these intrinsic alterations are not known; it is likely to be associated with the hyperglycemia and glycotoxicity present in the T2DM state. Support for this association comes from studies using ex vivo-created diabetic environments. In high glucose culture conditions, the proliferation and migration of healthy BMMSCs are attenuated, while apoptosis is increased due to the involvement of the TGFα p38 MAPK signaling pathway [25] or activation of the GSK3b-mediated cyclin D1 [26]. Such decreased proliferation and migration is restored by treatment with metformin, a first line antihyperglycemic agent which also activates AMPK (AMP-activated protein kinase) [27]. Moreover, studies conducted on BMMSCs harvested from T1DM (Type 1 diabetes mellitus) rats reported limited clonogenicity and/or cell proliferation [28,29,30,31] and increased apoptosis [32,33]. Hyperglycemia induces cell damage through ROS overproduction, mitochondrial dysfunction, and aberrant gene expression. It is the “common denominator” in the T1DM and T2DM murine diabetic models. Whereas the T1DM model is one of the insulin deficiency models, the ZDF-T2DM model is associated with insulin resistance and hyperinsulinemia conditions, which may have adverse effects on bone cells [20]. Dyslipidemia is also known to play an important role in BMMSCs’ fate [34]. Interestingly, in high-fat diet mice BMMSCs display a higher proliferation rate despite the increase in basal glucose levels [35]. In this regard, the use of hypoglycemic medications in the ZDF rat model could better clarify the role of chronic T2DM-associated hyperglycemia in BMMSC functions. For instance, in ZDF rats, empagliflozin, a SGLT2 inhibitor lowering glucose levels and the resulting glycotoxicity (oxidative stress, AGEs/RAGE signaling (advanced glycation end-products and their receptor)), prevents the development of endothelial dysfunction, despite persisting hyperlipidemia and hyperinsulinemia [36]. In this context, it will be of interest to study the effects of metformin—the most commonly prescribed hypoglycemiant drug, whose effects on MSC functionalities have been described from several origins [37]. However, it is challenging to ascertain the exact mechanisms behind hypoglycemiant drug effects because of their pleiotropic action.

Taken together, these data provide evidence that T2DM, even in an early stage, has detrimental effects on the BMMSCs which persist in cell culture, and that a return to normoglycemic conditions does not restore the “therapeutic” potential of the previously diabetic MSCs. The results obtained corroborate previous reports that diabetic stimuli, particularly hyperglycemia, induce an array of epigenetic modifications leading to a phenomenon called “metabolic memory” [33]. It is worth noting that epigenetic mechanisms include DNA methylation, histone modification, and various miRNA-mediated processes [38]. It has been reported that the profile of circulating miRNA is altered during the natural progression of T2DM, probably reflecting changes in diabetes-related tissues such as pancreas, liver, heart, skeletal muscle, and adipose in ZDF rats [39] and in other T2DM models [40]. Furthermore, growing evidence suggests that, in a T2DM context, the miRNA expression in MSCs is modified, as it has been shown recently that T2DM-adipose derived MSCs, showing reduced viability and proliferation, exhibit significant differences in the expression of miRNAs involved in cell proliferation (*miR-16-5p*, *miR-146a-5p*, and *miR-145-5p*), as well as miRNA and genes responsible for glucose homeostasis and insulin sensitivity (*miR-24-3p*, *140-3p*, *miR-17-5p*, *SIRT1*, *HIF-1α*, *LIN28*, *FOXO1*, and *TGFβ*) [41]. Further studies are needed to clarify T2DM-induced epigenetic changes in BMMSCs and their functionalities.

Furthermore, the results of the present study provided evidence for a differential potential of ZL- and ZDF-BMMSCs to differentiate into adipogenic and osteogenic lineages in vitro under normoglycemic conditions. Compared to ZL-BMMSCs, ZDF-BMMSCs exhibited enhanced adipogenesis, a phenomenon also observed in db/db mice [42], in WNIN/GR-Ob (mutant) rats [43] and in a patient with T2DM [44], suggesting that the BMMSCs removed from a hyperglycemic and hyperinsulinemic T2DM-state retained the enhanced adipogenic potential. Enhanced adipogenesis has also been shown in healthy BMMSCs cultured in medium complemented with sera isolated from T2DM patients [45], suggesting that the T2DM milieu plays a major role in inducing the differentiation of human BMMSCs. The mechanism behind T2DM-induced adipogenesis is not completely understood. Besides hyperglycemia-increased production of ROS and the modification of antioxidant mechanisms [14], the hyperinsulinemia-induced Nox4-derived oxidants [42] also lead to altered BMMSC differentiation. In the present study, ZDF-BMMSCs exhibited elevated levels of ROS, even when cultured under normoglycemic conditions, suggesting that T2DM milieu may induce permanent cellular alterations involved in ROS production, a condition which may also affect adipogenic differentiation of cells. The increased tendency of diabetic BMMSCs to differentiate into adipocytes is often associated with reduced osteogenic potential. The ZL- and ZDF-BMMSCs tested in the present study exhibited similar osteogenic differentiation. These results are in line with the reports on BMMSCs from an obese T2DM mouse model and from T2DM patients who exhibited increased adipogenic potential, without changing their osteogenic potential [44]. On the contrary, reduced osteogenic differentiation was mainly reported for BMMSCs derived from T1DM murine models [7,28,29,30,31]. The exact mechanism underlying this difference is not known. It may be the result of T1DM and T2DM effects on the biological aspects of BMMSCs, as well as the duration of T2DM. Undoubtedly, further studies are needed to elucidate if, and how, the insulinemia and hyperglycemia associated with T2DM may affect BMMSCs.

Concerns remain regarding the angiogenic potential of autologous MSCs in the T2DM environment [46]. The present study also investigated the proangiogenic effects of BMMSCs in the ZDF model. Pertinent results provided evidence for a defective angiogenic potential of ZDF-BMMSCs, lending support to those reported for BMMSCs derived from db/db mice [42] and for healthy BMMSCs pre-conditioned with serum from T2DM [47,48]. The decreased angiogenic potential of ZDF-BMMSCs is probably attributed to a dysfunctional signaling of endothelial cells arising from a disturbed balance between pro- and anti-angiogenic factors. Gene analysis of ZDF-BMMSCs cultured under normoglycemic conditions revealed the alteration of angiogenesis-related gene expression. In recent reports, the decreased angiogenic potential of BMMSCs was ascribed mainly to changes in the secretome composition [47,48]. In contrast to these studies, previous studies of our group [23] showed that secretome of ZDF-BMMSCs, implanted under similar conditions, resulted in increased angiogenesis, suggesting differences in the angiogenic effects between implanted cells and their secretome.

It is plausible that some limitations may have affected the present study. Firstly, in the ZDF rat model used, it is difficult to distinguish the effects of hyperglycemia from those of the obesity-associated dyslipidemia and hypertriglyceridemia. Secondly, in comparison to diet-induced polygenic models, the ZDF rat is a monogenic model in which leptin-signaling deficiency, accounted for the phenotype, does not reflect the complex background and physiopathology of T2DM [49]. On the other hand, in diet-induced polygenic models, used to study the interaction of diet and genes in obesity, “insulinoresistance” and T2DM complications, the choice of rodent strain, composition of high-fat formula, and combination treatment with streptozotocin are not yet standardized, thereby impairing practicability and comparability [50]. In the present study, the ZDF rat model was chosen because it mostly displays the metabolic, microvascular [4,22], and musculoskeletal features of T2DM reported in humans [21]. Moreover, results from our group revealed an altered composition of the secretome and the paracrine effects of BMMSCs [23].

In summary, the present study provided evidence that BMMSCs exhibit impaired functionality ex vivo under normoglycemic conditions and/or after their transplantation in a non-diabetic environment. These data highlight the fact that BMMSCs harvested even from the short-term T2DM environment exhibit changes which make them dysfunctional in situ and limit their therapeutic potential post-transplantation. The present study further emphasizes the need for further research and/or new approaches to restore the functions of defective MSCs isolated from diabetic subjects.

## Data Availability

The data presented in this study are available on request from the corresponding author.
